# Latent profile analysis of the symptoms for posttraumatic stress disorder and psychological resilience in Chinese adolescents experiencing post Covid-19: a quantetative study

**DOI:** 10.1186/s40359-026-03987-8

**Published:** 2026-04-07

**Authors:** Lu Zhou, Yinsong Sun, Runli Yang, Qinghua Zhao, Mingzhao Xiao

**Affiliations:** 1 International Medical Center, The First Affiliated Hospital of Chongqing Medical University, Chongqing, China; 2 Department of Orthopedics, The First Affiliated Hospital of Chongqing Medical University, Chongqing, China; 3 Department of Urology, The First Affiliated Hospital of Chongqing Medical University, Chongqing, China; 4 Department of Nursing, Chongqing Nursing Vocational College, Chongqing, China; 5 Nursing Research Center, The First Affiliated Hospital of Chongqing Medical University, No. 24, Shiyou Road, Yuzhong District, Chongqing City, 400000 China

**Keywords:** Long COVID, Latent profile analysis, PTSD, Resilience, Mental health

## Abstract

**Objectives:**

This study aimed to identify distinct, person-centered profiles of posttraumatic stress disorder (PTSD) symptoms and psychological resilience among Chinese adolescents experiencing post-COVID-19, and to explore factors associated with profile membership.

**Methods:**

A cross-sectional study was conducted from March to May 2023, and a total of 932 adolescents were recruited from Chongqing in China. Participants completed the Posttraumatic Stress Disorder Checklist-Civilian Version (PCL-C) and the Connor-Davidson Resilience Scale (CD-RISC). To assess and control for potential common method bias, the common latent factor (CLF) method was employed within a confirmatory factor analysis framework. Latent profile analysis (LPA) was used to identify homogeneous subgroups based on PTSD and resilience scores. Differences in scale scores across the resulting profiles were examined using one-way ANOVA, with post-hoc pairwise comparisons conducted using the Bonferroni correction to control Type I error. Multinomial logistic regression analysis was used to explore predictors associated with membership in the different latent profiles.

**Results:**

Among 932 adolescents experiencing long COVID, PTSD-resilience models showed five latent profiles: "low resilience-medium PTSD" (10.63%), "moderately low resilience-moderately high PTSD" (6.77%), "medium resilience-moderately low PTSD" (27.61%), "moderately high resilience-high PTSD" (30.93%), and "high resilience-low PTSD" (24.06%). Women gender, number of COVID-19 vaccinations, and fear of severe reinfection were significantly associated with profile membership.

**Conclusions:**

Our research presents the first report on the intricate coexistence patterns between PTSD and resilience among adolescents, significantly expanding our understanding of the protective factors for adolescent mental health. As a crucial buffering mechanism against PTSD, resilience underscores the urgency for every citizen in the post-pandemic era to identify adolescents who may be at high risk of PTSD and/or exhibit low resilience, and to promptly initiate targeted therapeutic interventions and rehabilitation efforts.

**Supplementary Information:**

The online version contains supplementary material available at 10.1186/s40359-026-03987-8.

## Introduction

In the aftermath of the COVID-19 pandemic, although it is no longer considered an immediate emergency, its profound and enduring implications on individuals' and communities' lives worldwide are undeniable [24]. Starting in 2023, China has gradually relaxed its policies, with lockdown measures being progressively lifted. A persistent clinical challenge brought to the fore is the high prevalence of post-COVID-19 condition, commonly referred to as Long COVID. This syndrome is characterized by a constellation of symptoms, including fatigue, cognitive dysfunction, and sleep disturbances, which can persist for weeks to months beyond the acute phase of infection, exerting a protracted impact on a substantial proportion of patients [19, 34, 41, 44, 50]. For children and adolescents, the most prevalent manifestations of Long COVID are frequently emotional and psychological in nature [33]. Consequently, the global COVID-19 pandemic can be regarded as a traumatic stressor, exerting a significant impact on mental health, particularly among adolescents, and potentially precipitating the development of Post-Traumatic Stress Disorder (PTSD) [5, 45].

PTSD represents one of the most prototypical and prevalent adverse psychological outcomes following traumatic exposure. A systematic review indicates that its prevalence rate during the COVID-19 pandemic reached up to 26.9% [9]. Adolescents appear to be particularly vulnerable to such psychological sequelae, exhibiting a higher susceptibility to PTSD and other emotional disorders compared to adults—a vulnerability exacerbated by fears of illness or death, pandemic-related uncertainty, and challenges in adapting to remote learning [28, 37, 54]. Notably, even among those with relatively intact physical health, suboptimal psychological functioning has been commonly observed [22], with studies consistently reporting varying severity of PTSD symptomatology in affected populations [3] 

The trajectory of adolescent mental health during the pandemic has been dynamic. While significant deterioration was noted in the initial phases, some stabilization occurred later, attributed in part to enhanced resilience, vaccination uptake, and the resumption of social interactions [35]. Nevertheless, emotional responses have varied widely, with some adolescents experiencing improved affect between pandemic waves, whereas others—particularly those with higher sensitivity—have endured a progressive increase in negative emotions over time [11]. Importantly, emerging evidence links persistent post-COVID somatic symptoms (PCS), defined as the persistence of symptoms such as fatigue, dyspnea, and cognitive problems for at least 12 weeks after acute COVID-19, with sustained elevations in depressive and PTSD symptoms, detectable up to 9–26 months post-infection [21]. Despite these insights, research focusing specifically on PTSD among adolescents in the post-pandemic phase remains notably scarce, underscoring a critical gap in the current literature.

Psychological resilience, a positive and process-oriented defense mechanism, stems partly from meaningful, supportive, and functional social networks [13]. This multidimensional construct becomes particularly evident when an individual encounters adversity or traumatic events, functioning not only as a protective factor against trauma exposure but also mitigating the development of psychopathological symptoms [39]. Notably, the pandemic experience may paradoxically foster resilience in some individuals, enhancing their capacity to cope with subsequent stressors. This underscores the imperative to understand not just risk, but also the factors that promote adaptive outcomes. The heterogeneous psychological responses to COVID-19, as evidenced by numerous studies, underscore the importance of targeted prevention and intervention efforts for high-risk population [51].

Despite the easing of lockdown restrictions, overall physical and mental health in the general population has shown a concerning decline since the onset of the pandemic [31], pointing to its persistent and pervasive impact. Prior research on adolescent PTSD and resilience, primarily conducted during the COVID-19 pandemic [2, 6, 26, 36, 55], or through related longitudinal studies [40, 48], have largely adopted variable-centered approaches. While such frameworks yield valuable insights into general trends, they often overlook the critical role of individual differences in shaping heterogeneous psychological outcomes. In response, this study introduces a person-centered, latent profile analysis (LPA) method, aiming to classify adolescents probabilistically into distinct subgroups based on their combined PTSD and resilience characteristics. This method allows for a nuanced examination of within-population heterogeneity [18] and moves beyond blanket assumptions of population uniformity.

Given the scarcity of research examining the co-occurrence of PTSD and psychological resilience among adolescents, particularly within the post-pandemic, multiple waves of COVID-19, this study focuses on the co-evolution and interaction dynamics. We aim to elucidate how resilience may function as a psychological buffer against PTSD in this unique clinical and developmental context. By analyzing the psychophysiological correlates, our findings seek to inform future prevention initiatives and contribute to the development of targeted, effective psychological interventions tailored specifically for adolescents experiencing Long COVID.

## Materials and methods

### Study design

This cross-sectional descriptive study was designed and reported in accordance with the Strengthening the Reporting of Observational Studies in Epidemiology (STROBE) guidelines (Supplementary 1).

### Study population

Nine hundred thirty two adolescents from Chongqing Nursing Vocational College in China were recruited as study participants between March and May 2023 using a simple random sampling method. The participants were aged 18–21 years, which aligns with the World Health Organization (WHO) definition of adolescents (10–24 years old) and the demographic characteristics of students at the recruited institution [38] (where the majority of students are in the late adolescent period, transitioning from adolescence to young adulthood). The inclusion criteria were as follows: (1) 18 years of age or older, (2) voluntary participation, (3) conscious and able to communicate effectively, and (4) absence of a diagnosed mental illness. The exclusion criteria are as follows: (1) mental illness, intellectual disability, or severe physical illness; (2) leave of absence from work or school for more than one month.

### Procedure

This study was pre-surveyed with 30 participants in January 2023 to assess the comprehensibility of the online self-report questionnaire and to identify any technical issues. From March to May 2023, investigators distributed the online questionnaire via Questionstar (https://www.wjx.cn/). Participants completed the questionnaire by accessing the WeChat quick response code or link provided. All questions were mandatory, and a time limit of 20 min was set for completion.

### Measurements

#### General information questionnaire

A self-designed general information questionnaire was used to collect participants' demographic data, and disease diagnosis sections including gender, age, grade, education level and type of physical activity. To ensure its relevance and applicability, the physical symptoms section was adapted from the Global COVID-19 Clinical Platform Case Report Form (CRF) for Post-COVID Condition (Post COVID-19 CRF), tailoring it to the unique characteristics of the study population. Table 1 provides an overview of some of the variable definitions and codes used in the questionnaire.

**Table 1:** Case of variable assignment

Variable	Assignment mode
Gender	Men = 1; women = 2
Age	18 = 1; 19 = 2; 20 = 3; 21 = 4;22 = 5;23 = 6
BMI	< 18.5 = 1; 18.5 ~ 23.9 = 2; 24 ~ 27.9 = 3; ≥ 28 = 4
Grade	Measured value
Situation of infection	Symptomatic, + = 1; symptomatic, -/not tested = 2; asymptomatic, + = 3; asymptomatic, -/not tested = 4
Number of vaccinations	0 = 1; 1 = 2; 3 = 3; 4 = 4; > 4 = 5; none = 6
Primary assay in this infection	Antigen reagents = 1; nucleic acid test = 2; antigen reagent + nucleic acid test = 3
Symptoms (fatigue, weakness, memory loss, constipation/diarrhea, anxiety/depression, dizziness, slow movement, sleep disorders (less/more sleep), difficulty concentrating, joint pain, dry cough, nausea and vomiting, loss of interest, headache, muscle pain, weakness in the limbs, loss of appetite, hearing impairment, swallowing problems, olfactory disturbances, taste disorders)	Never = 0; Had, persistent, intermittent, disappeared = 1
Anxiety situation	Yes = 1; no = 0;
Time and amount of learning situation	Yes = 1; no = 0;
WHO-5	Measured value
PCL-C	Measured value
CD-RISC	Measured value

*PCL-C* Posttraumatic stress disorder checklist-civilian version, *CD-RISC* Connor-davidson resilience scale, *WHO-5* World health organization five-item well-being index

#### COVID-19 negative emotiont situation scale

It was developed based on a systematic review of domestic and international literature, supplemented by consultations with experts in respiratory medicine, psychiatry from a tertiary hospital in Chongqing, and nursing education from vocational colleges. Four core domains of COVID-19-related negative emotional impact were conceptualized: anxiety situation, negative impacts on learning (both personal and environmental), and time and amount of learning situation (18 items total; see Supplementary Fig S1 for the confirmatory factor analysis model structure).

An initial item pool was generated and evaluated by ten qualified experts, each with at least five years of professional experience or a master's degree or above. Content validity was supported by a content validity ratio (CVR) of 0.60 and an expert authority coefficient of 0.925. A pre-validation survey was administered to 50 participants (not included in the final sample) to refine items for clarity and relevance. Detailed psychometric properties of the final scale are provided in Supplementary Table S1. (1) Reliability: The Kaiser–Meyer–Olkin (KMO) measure was 0.840. Cronbach's α coefficients for the total scale and dimensions ranged from 0.864 to 0.945. Composite reliability (CR) for each dimension ranged from 0.812 to 0.924. (2) Validity: confirmatory factor analysis (CFA) supported the hypothesized four-factor structure (Supplementary Fig S1). All standardized factor loadings were significant (*p* < 0.001). The average variance extracted (AVE) for each dimension ranged from 0.636 to 0.746. Model fit indices were as follows: χ^2^/df = 13.692, CFI = 0.879, NFI = 0.871, IFI = 0.880, RMSEA = 0.003 (95% CI: 0.002–0.005).

#### World Health Organization five-item well-being index (WHO-5)

The WHO-5, a validated instrument developed by the World Health Organization, was employed to assess the participants' subjective well-being over the past two weeks. Comprising five items, the questionnaire utilizes a 6-point Likert scale ranging from 0 ("never") to 5 ("all the time"), yielding a total score between 0 and 30. Higher scores indicate higher levels of well-being, with a score of ≤ 13 suggesting possible depression or reduced quality of life. The instrument has consistently demonstrated good internal consistency, with a Cronbach's alpha coefficient ranging from 0.83 to 0.92 [23]. In this study, the WHO-5 achieved a Cronbach's alpha of 0.922, confirming its reliability.

#### Posttraumatic Stress Disorder Checklist-Civilian Version (PCL-C)

The PCL-C, a widely used instrument for assessing PTSD symptoms, was adopted for this study based on the criteria outlined in the Diagnostic and Statistical Manual of Mental Disorders, Fourth Edition (DSM-IV) [47]. It consisted of 17 items, including 3 dimensions of re-experiencing (5 items), avoidance (7 items), and hypervigilance (5 items), with each item using a 5-point scale from 1 to 5, with 1 = no reaction, 2 = mild reaction, 3 = moderate reaction, 4 = severe reaction, and 5 = very severe reaction, for a total score of 17–85. The scale has good reliability and validity, with Cronbach α coefficient of 0.910 [30]. A total score of 38 or higher is considered indicative of PTSD [10, 15], and the presence of a symptom can be confirmed if the score for each item exceeds 3, in accordance with DSM-W criteria. In this study, the PCL-C demonstrated a Cronbach's alpha of 0.935, underscoring its reliability.

#### Connor-Davidson resilience scale (CD-RISC)

The CD-RISC, a well-established instrument for measuring psychological resilience was developed by Connor and Davidson [7], and was translated into Chinese by Wang et al. [46]. he scale comprises 25 items organized into three dimensions: resilience (items 11–23), strength (items 1, 5, 7–10, 24, 25), and optimism (items 2–4, 6). Participants respond on a 5-point Likert scale ranging from 0 ("not at all") to 4 ("almost always"), resulting in a total score ranging from 0 to 100. Higher scores indicate greater psychological resilience. The CD-RISC has consistently demonstrated good psychometric properties, with a Cronbach's alpha coefficient of 0.890 [52]. In this study, the internal consistency reliability of the dimensions was found to be 0.940, further validating the scale's applicability and reliability.

### Ethical considerations

Prior to participation, all participants were presented with an electronic informed consent form detailing the study purpose, procedures, potential risks and benefits, data usage, confidentiality measures, and their right to withdraw at any time without penalty. Informed consent was confirmed by clicking an "Agree" button, and participants could exit the questionnaire at any time before submission without penalty. All collected data were encrypted and stored on the secure server of the Questionstar platform, with the access restricted to one designated member of the research team who held the password. Other team members could only obtain data access by submitting a formal request to the password custodian. In accordance with ethical guidelines, raw data will be retained for five years after publication and then permanently deleted. Participants who scored ≤ 13 on the WHO-5 or ≥ 38 on the PCL-C were provided with a tailored psychological support resource sheet, including contact information for the college counseling center and local mental health services. Follow-up reminders were sent to these participants to encourage utilization of support services. The study protocol was reviewed and approved by the Ethics Committee of the First Affiliated Hospital of Chongqing Medical University (approval number: 2023–334).

### Statistical analysis

#### Common method deviation test

Given the risk of common method bias arising from the collection of all latent variables through self-reported data from the same respondents, the common latent factor (CLF) medthod [1] was employed to ensure measurement model reliability and result validity, with all model estimations performed using AMOS 24.0. This method involves introducing an additional latent factor (CLF) into the baseline confirmatory factor analysis (CFA) model, linking it to all observed indicators to capture systematic variance potentially attributable to the common data source. The statistical significance of method bias was then assessed by comparing the model fit indices before and after including the CLF via a chi-square difference test. Common method bias is considered non-critical if the variance explained by the method factor is below the 40% threshold and if the maximum absolute change in any standardized factor loading after controlling for the CLF is less than 0.20.

#### Latent profile analysis

To uncover latent subgroups within our sample based on PTSD symptoms and psychological resilience, we utilized latent profile analysis (LPA) implemented in Mplus 8.3. This approach employs a latent profile model (LPM) to identify distinct profiles within the population, focusing on continuous variables [16, 29].

We initiated the LPA by fitting a single-category model and iteratively increased the number of categories, evaluating each model based on fit indices such as the Akaike Information Criterion (AIC), Bayesian Information Criterion (BIC), Sample Size Corrected BIC (αBIC), entropy, and likelihood ratio tests (including the Lo-Mendell-Rubin (LMR) test, Vuong-Lo-Mendell-Rubin (VLMR) test, and Bootstrap Likelihood Ratio Test (BLRT) [42]. Smaller AIC, BIC, and αBIC values indicate superior model fit, while higher entropy values suggest more precise classification. Significant *p*-values for the LMR, VLMR, and BLRT indicate potential improvement in model fit with additional categories. Additionally, we considered model parsimony (preferring less complex models) and profile size (ensuring each profile represents at least 5% of the total sample to avoid non-replicable profiles) [32].

#### Descriptive analyses

Using SPSS 26.0, data from different sub-class were described with mean ± standard deviation $\overline x\;\pm s$ for quantitative data and frequency and composition ratio for count data.

#### Single factor and multi-factor analysis

After identifying the optimal Latent Profile Analysis (LPA) model and classifications, intergroup comparisons were conducted. For continuous variables, Student's t-test was employed for comparisons between two groups, and Analysis of Variance (ANOVA) was utilized for comparisons involving more than two groups. For categorical variables, Chi-square (χ^2^) test was adopted. To control the Type I error risk arising from multiple comparisons, post‑hoc tests were performed using Bonferroni correction, maintaining the family‑wise error rate (FWER) at α = 0.05. Variables that demonstrated a statistically significant association (*P* < 0.05) in these univariate analyses were selected as candidate predictors for the subsequent multivariable model. To address multicollinearity among the screened candidate variables, the variance inflation factor (VIF) was examined, with a VIF < 10 adopted as the criterion for indicating no severe multicollinearity; variables with VIF > 10 were removed. After adjusting for potential confounders, a multivariate logistic regression model was used to evaluate the relationships between the identified latent profiles and the retained relevant variables, thereby analyzing the strength of association between various factors and membership in different subtypes. Adjusted Odds Ratios (AORs) and their 95% Confidence Intervals (95% CI) were calculated to quantify the associations. All tests were conducted using two-sided methods, with a significance level set at 0.05.

## Results

### Data completeness and missing value handling

A total of 932 adolescents were included in the final analysis from an initial pool of 1100 eligible individuals. Prior to analysis, data completeness was confirmed. The online questionnaire was designed with mandatory response fields (via the Questionstar platform) to prevent missing data. A systematic review confirmed no missing values in key variables (PCL-C, CD-RISC, demographics), as submission required completion of all items. Therefore, no data imputation was necessary. Exclusions from the initial pool were based on the following quality-control criteria: 31 participants who completed the survey in less than 2 min, 56 with patterned responses indicating inattentiveness, and 81 who reported no post-COVID symptoms during the study period.

### Common method deviation test

Common method bias was assessed using the CLF approach. The model fit indices before and after adding the CLF are summarized in Supplementary Table S2. The chi-square difference between the original model (χ^2^ = 2806.676, df = 222) and the model with the CLF (χ^2^ = 2763.629, df = 258) was not significant (Δχ^2^ = 43.047, Δdf = 36, *P* > 0.05), indicating no statistically significant improvement in fit after including the method factor. The variance explained by the method factor (32.7%) and the maximum absolute change in standardized factor loadings (0.12) were both well below their respective critical thresholds of 40% and 0.20. Furthermore, the average variance explained by substantive constructs (0.805) substantially exceeded the average method variance (0.016), with a ratio of approximately 50:1 (see Supplementary Table S3 for details). Collectively, these results indicate that significant common method bias is not present, the measurement model is reliable, and it can be used for subsequent hypothesis testing in the structural model.

### Results of latent profile analysis

Table 2 shows that for the one to six-profile models. Specifically, while profile6 has the most minor and statistically significant LL, AIC, BIC, and aBIC, there is 1 category in profile 6 that accounts for less than 5% (0.04), for the sake of model metrics and simplicity, and in light of the risk that the model is not replicable. In addition, profile 4's LL, AIC, BIC, aBIC, and entropy values were not as good as profile 5's. Therefore, we chose the profile 5 solution as the optimal model. Supplementary Table S4 showed the attribution probability matrix for the 5 potential profiles. The average probability of attribution of each class to its corresponding potential profile ranged from 89.8% to 93.5%, indicating that the results of the model for the five potential profiles in this study were plausible.

**Table 2:** Model fit indexes of latent profile analysis (*n* = 932)

**Model**	**K**	**Log–likelihood**	**AIC**	**BIC**	**aBIC**	**Entropy**	LMR (*p*_value)	BLRT (*p*_value)	**Probabilities of profiles**
One-profile	12	−18,379.492	36,782.984	36,841.019	36,802.908	—	—	—	—
Two-profile	19	−17,893.807	35,825.615	35,917.504	35,857.161	0.833	0.000	0.000	0.56/0.45
Three-profile	26	−17,530.155	35,112.31	35,238.053	35,155.479	0.832	0.000	0.000	0.36/0.29/0.35
Four-profile	33	−17,354.581	34,775.162	34,934.759	34,829.954	0.848	0.000	0.000	0.30/0.26/0.32/0.12
Five-profile	40	−17,200.398	34,480.795	34,674.245	34,547.209	0.864	0.0236	0.000	0.24/0.31/0.28/0.11/0.09
Six-profile	47	−17,080.496	34,254.991	34,482.295	34,333.028	0.870	0.0299	0.000	0.08/0.17/0.29/0.29/0.13/0.04

*K* Number of free parameters, *AIC* Akaike information criterion, *BIC* Bayesian information criterion, *αBIC* Same-size adjusted Bayesian information criterion, *LMR* Lo-mendell-rubin likelihood ratio test, *VLMR* Vuong-lo-mendell-rubin likelihood ratio test, *BLRT* Bootstrapped likelihood ratio test, —Not applicable

### Naming of latent profile

**Fig. 1:**
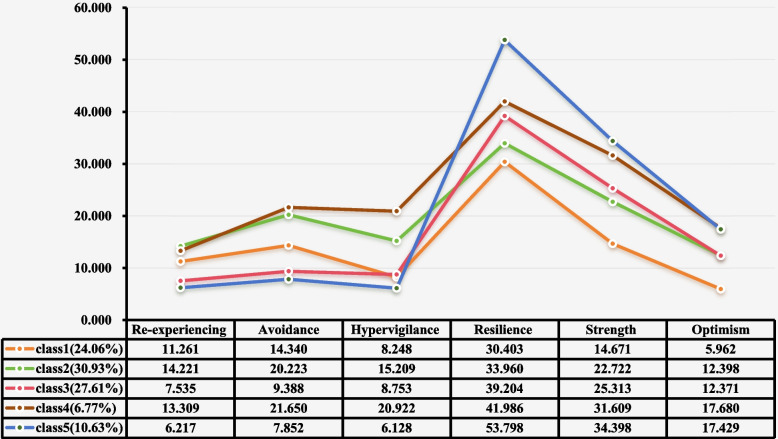
Different classes of the PCL-C and CD-RISC among adolescents

Figure 1 illustrates the scores of five latent profiles of mental health symptoms among Chinese adolescents experiencing Long COVID-19 across six dimensions. Class 5 scored significantly lower than all other classes on all three dimensions of PCL-C but higher on all three dimensions of resilience, hence labeled "High Resilience-Low PTSD" (*n* = 99, 10.63%). Class 4 exhibited the highest scores on PCL-C dimensions among all profiles, ranking second highest on resilience dimensions, thus named "Moderately High Resilience-High PTSD" (*n* = 64, 6.77%). Class 3 scored between Classes 1 and 5 on PCL-C dimensions and second only to Class 4 on resilience, categorized as "Medium Resilience-Moderately Low PTSD" (*n* = 257, 27.61%). Class 2 scored between Classes 4 and 1 on PCL-C dimensions, second only to Class 3 on resilience, designated "Moderately Low Resilience-Moderately High PTSD" (*n* = 288, 30.93%). Class 1 scored between Classes 2 and 3 on PCL-C dimensions, with lower resilience scores than the other four classes, labeled "Low Resilience-Medium PTSD" (*n* = 224, 24.06%).

### Demographic information and inter-profile characteristic differences

A total of 932 adolescents were included in the data analysis, having been selected from an initial pool of 1100 adolescents who met the inclusion and exclusion criteria. Exclusions comprised 31 adolescents who completed the questionnaire within 2 min, 56 with consistent responses indicating potential inattentiveness, and 81 with no reported post COVID-related symptoms during the survey period. The sample consisted of 565 women (60.62%), with a mean age of 19.29 ± 0.956 years. The number of COVID-19 vaccinations, the distribution was: 1 dose (*n* = 90, 9.66%), 2 doses (*n* = 447, 47.96%), and 3 doses (*n* = 395, 42.38%).

The results of the analysis of variance using the χ^2^ test showed that gender, the number of vaccinations, symptoms of weakness, hearing impairment, olfactory impairment, and taste impairment, neither study nor times no change or both decreased, and fear of being re-infected in a more severe form, developing Post-acute sequelae of SARS-CoV-2 (PASC), social isolation/decrease in social relationships/worsening of friendships, family finances become difficult, academic delays, and nothing to worry about were statistically significant (*p* < 0.05). The differences in the remaining categories were not statistically significant (*P* > 0.05). See Table 3 for details.

**Table 3:** Disease demographics and study-related characteristics by potential file membership, (*n* = 932)

Variable	Class 1(%)	Class 2(%)	Class 3(%)	Class 4(%)	Class 5(%)	Full sample (%)	χ^2^/F	*P*
Gender							44.547	0.000 *
Men	107a(47.77)	137a(47.57)	77b, c(29.96)	27a, b(42.19)	19c(19.19)	367(39.38)		
Women	117a(52.23)	151a(52.43)	180b, c(70.04)	37a, b(57.81)	80c(80.81)	565(60.62)		
Age							14.224	0.287
18	44a(19.64)	49a(17.01)	52a(20.23)	13a(20.31)	13a(13.12)	171(18.35)		
19	88a(39.29)	122a(42.36)	126a(49.03)	25a(39.06)	46a(46.46)	407(43.67)		
20	75a(33.48)	86a(29.86)	59a(22.96)	22a(34.38)	39a(30.30)	272(30.15)		
21	17a(7.59)	31a(10.76)	20a(7.78)	4a(6.25)	10a(10.12)	73(7.83)		
BMI							6.66	0.879
<18.5	70a(31.25)	80a(27.78)	72a(28.02)	19a(29.69)	31a(31.31)	272(29.18)		
18.5 ~ 23.9	4a(1.79)	10a(3.47)	6a(2.33)	0a(0)	1a(1.01)	21(2.25)		
24 ~ 27.9	134a(59.82)	176a(61.11)	159a(61.87)	42a(65.63)	62a(62.63)	573(61.48)		
≥ 28	16a(7.14)	22a(7.64)	20a(7.78)	3a(4.69)	5a(5.05)	66(7.09)		
Grade							9.176	0.057
1	133a(59.38)	172a(59.72)	181a(70.43)	37a(57.81)	63a(63.64)	586(62.88)		
2	91a(40.63)	116a(40.28)	76a(29.57)	27a(442.19)	36a(36.36)	346(37.12)		
Number of vaccinations							49.87	0.000 *
1	22a, b(9.82)	37b(12.85)	22a, b(8.56)	7a, b(10.94)	2a(2.02)	90(9.66)		
2	121a(54.02)	162a(56.25)	100b(38.91)	29a, b(45.31)	35b(35.35)	447(47.96)		
3	81a(36.16)	89a(30.9)	135b(52.53)	28a, b(43.75)	62b(62.63)	395(42.38)		
Primary assay in this infection							6.956	0.541
Antigen reagents	152a(67.86)	182a(63.19)	158a(87.78)	46a(71.88)	70a(70.71)	608(65.24)		
Nucleic acid test	33a(14.73)	48a(16.67)	49a(19.07)	11a(17.19)	14a(14.14)	155(16.63)		
Antigen reagent + nucleic acid test	39a(17.41)	58a(20.14)	50a(19.46)	7a(10.49)	15a(15.15)	169(18.13)		
Fatigue							34.419	0.001*
Had	42a(18.75)	46a(15.97)	50a(19.46)	14a(21.88)	11a(11.11)	163(17.49)		
Persistent	16a, b(7.14)	9b(3.13)	35a(13.62)	3a, b(4.69)	16a(16.16)	79(8.48)		
Intermittent	147a, b(65.63)	209b(72.57)	153a(59.53)	40a, b(62.50)	64a, b(64.65)	613(65.77)		
Never	19a(8.48)	24a(8.33)	19a(7.39)	7a(10.94)	8a(8.08)	77(8.26)		
Weakness							119.046	0.000 *
Had	28a(12.50)	14b(4.86)	64c, d(25.00)	7a, b, c(10.49)	32d(32.32)	145(15.56)		
Persistent	19a(8.48)	47a(16.32)	22a(8.59)	12a(18.75)	0b(0)	100(10.73)		
Intermittent	135a(60.27)	177a(61.46)	164a(63.67)	36a(56.25)	62a(62.63)	573(61.59)		
Never	42a(18.75)	50a(17.36)	7b(2.73)	9a, c(14.06)	5b, c(5.05)	113(12.12)		
Memory loss							117.029	0.000 *
Had	11a(4.91)	12a(4.17)	33b(12.84)	6a, b(9.38)	19b(19.19)	81(8.69)		
Persistent	23a(10.27)	25a(8.68)	76b(29.57)	7a(10.94)	15a, b(15.15)	146(15.67)		
Intermittent	137a(61.16)	180a(62.5)	131a(50.97)	32a(50.00)	56a(56.57)	536(57.51)		
Never	53a(23.66)	71a(24.65)	17b(6.61)	19a(29.69)	9b(9.09)	169(18.13)		
Constipation/diarrhoea							55.822	0.000 *
Had	27a(12.05)	26a(9.03)	55b(21.40)	14a, b(21.88)	8b(8.08)	130(13.95)		
Persistent	10a(4.46)	15a(5.21)	10a(3.89)	8b(12.50)	0a(0)	43(4.61)		
Intermittent	134a(59.82)	170a(59.03)	165a(64.20)	24b(37.50)	85a(85.86)	578(62.02)		
Never	53a(23.66)	77a(26.74)	27a(10.51)	18a(28.13)	6a(6.06)	181(19.42)		
Anxiety/depression							51.741	0.000 *
Had	14a(6.25)	29a(10.07)	31a(12.06)	4a(6.25)	14a(14.14)	92(9.87)		
Persistent	22a(9.82)	34a(11.81)	19a(7.39)	9a(14.06)	7a(7.07)	91(9.76)		
Intermittent	151a, b, c(67.41)	162c(56.25)	183b(71.21)	32a, c(50.00)	77b(77.78)	605(64.91)		
Never	37a, b, c(16.52)	63c(21.88)	24b, d(9.34)	19a, c(29.69)	1d(1.01)	144(15.46)		
Dizziness							68.269	0.000 *
Had	28a, b, c(12.50)	30c(10.42)	49a, b(19.07)	3a, c(4.69)	23b(23.23)	133(14.27)		
Persistent	10a(4.46)	12a(4.17)	19a(7.39)	3a(4.69)	6a(6.06)	50(5.36)		
Intermittent	161a(71.88)	190a(65.97)	181a(70.43)	48a(75.00)	70a(70.71)	650(69.74)		
Never	25a(11.16)	56a(19.44)	8b(3.11)	10a(15.63)	0b(0)	99(10.63)		
Slow movement							46.855	0.000 *
Had	21a, b, c(9.38)	23c(7.99)	36a, b, c(14.01)	2a, c(3.13	19b(19.19)	101(10.84)		
Persistent	22a(9.82)	29a(10.07)	18a, b(7.00)	6a, b(9.38)	1b(1.01)	76(8.15)		
Intermittent	144a, b(64.29)	153b(53.13)	144a, b(56.03)	48a(75.0)	67a, b(67.68)	556(59.66)		
Never	37a(16.52)	83b(28.82)	59a, b(22.96)	8a, b(12.50)	12a(12.12)	199(21.35)		
Sleep disorders (less/more sleep)							47.337	0.000 *
Had	16a(7.14)	31a(10.76)	33a(12.84)	1a(1.56)	14a(14.14)	95(10.19)		
Persistent	28a(12.50)	33a(11.46)	38a(14.79)	12a(18.75)	17a(17.17)	128(13.73)		
Intermittent	163a(72.77)	188a(65.28)	180a(70.04)	37a(57.81)	64a(64.65)	633(67.81)		
Never	17a, b(7.59)	36b, c(12.50)	6a(2.33)	14c(21.88)	4a, b(4.04)	77(8.27)		
Difficulty concentrating							64.363	0.000 *
Had	27a, b(12.05)	25b, c(8.68)	42a, b(16.34)	0c(0)	20a(20.20)	114(12.23)		
Persistent	11a(4.91)	15a(5.21)	23a, b(8.95)	10b(15.63)	4a, b(4.04)	63(6.76)		
Intermittent	143a(63.84)	208a(72.22)	173a(67.32)	41a(64.06)	75a(75.76)	640(68.67)		
Never	43a(19.20)	40a, b(13.89)	19b, c(7.39)	13a(20.31)	0c(0)	115(12.34)		
Joint pain							102.028	0.000 *
Had	27a, b(12.05)	44a, b(15.28)	72c(28.02)	2a(3.13)	23b, c(23.23)	168(18.03)		
Persistent	12a(5.36)	22a(7.64)	1b(0.39)	5a(7.81)	2a, b(2.02)	42(4.51)		
Intermittent	168a(75.00)	168b(58.33)	169a, b(65.76)	41a, b(64.06)	74a(74.75)	620(66.52)		
Never	17a(7.59)	54b(18.75)	15a, c(5.84)	16b(25.00)	0c(0)	102(10.94)		
Dry cough							89.305	0.000 *
Had	19a(8.48)	19a(6.60)	56b, c(21.79)	6a, b(9.38)	31c(31.31)	131(14.06)		
Persistent	25a(11.16)	26a, b(9.03)	10b(3.89)	8a, b(12.50)	3a, b(3.03)	72(7.73)		
Intermittent	146a(65.18)	181a(62.85)	169a(65.76)	39a(60.94)	62a(62.63)	597(64.06)		
Never	34a, b(15.20)	62b(21.50)	22a, c(8.60)	11a, b(17.19)	3c(3.00)	132(14.15)		
Nausea and vomiting							40.892	0.000 *
Had	31a, b, c(13.84)	21c(7.29)	42a, b(16.34)	2a, c(3.13)	18b(18.18)	114(12.23)		
Persistent	11a(4.91)	18a(6.25)	6a(2.33)	4a(6.25)	0a(0)	39(4.18)		
Intermittent	152a(67.86)	199a(69.10)	185a(71.98)	42a(65.63)	71a(71.72)	649(69.64)		
Never	30a, b(13.39)	50a, b(17.36)	24b(9.34)	16a(25.00)	10a, b(10.10)	130(13.95)		
Loss of interest							42.077	0.000 *
Had	22a(9.80)	19a(6.59)	32a(12.50)	2a(3.13)	10a(10.10)	85(9.12)		
Persistent	11a(4.91)	16a(5.56)	12a(4.69)	6a(9.38)	1a(1.01)	46(4.94)		
Intermittent	155a(69.20)	183a(63.47)	177a(69.14)	42a(65.63)	85b(85.86)	642(68.88)		
Never	36a, b(16.09)	70b(24.38)	36a(13.67)	14a, b(21.88)	3c(3.03)	159(17.06)		
Headache							35.401	0.000 *
Had	19a(8.48)	25a(8.68)	32a(12.45)	6a(9.38)	12a(12.12)	94(10.09)		
Persistent	4a(1.79)	9a(3.13)	4a(1.56)	3a(4.69)	0a(0)	20(2.15)		
Intermittent	171a, b, c(76.34)	190c(65.97)	190a, b, c(73.93)	38a, c(59.38)	81b(81.82)	670(71.89)		
Never	30a, b, c(13.39)	64c(22.22)	31b(12.06)	17a, c(26.56)	6b(6.06)	148(15.87)		
Muscle pain							55.869	0.000 *
Had	16a(7.14)	40a, b(13.89)	42b(16.34)	9a, b(14.06)	13a, b(13.13)	120(12.88)		
Persistent	12a(5.36)	8a, b(2.78)	1b(0.39)	1a, b(1.56)	0a, b(0)	22(2.36)		
Intermittent	178a(79.46)	182b(63.19)	186a, b(72.37)	40b(62.50)	81a(81.82)	667(71.56)		
Never	18a(8.04)	58b(20.14)	28a, c(10.89)	14b, c(21.88)	5a(5.05)	123(13.2)		
Weakness in the limbs							54.448	0.000 *
Had	32a, b(14.29)	23b(7.99)	42a(16.34)	12a, b(18.75)	13a, b(13.13)	122(13.09)		
Persistent	15a(6.70)	18a(6.25)	8a(3.11)	0a(0)	0a(0)	41(4.40)		
Intermittent	145a(64.73)	187a(64.93)	189a, b(73.54)	41a, b(64.06)	82b(82.83)	644(69.10)		
Never	32a, b(14.29)	60b(20.83)	18a(7.00)	11a, b(17.19)	4a(4.04)	125(13.41)		
Loss of appetite							49.368	0.000 *
Had	18a(8.04)	27a(9.38)	48b(18.68)	8a, b(12.50)	9a, b(9.09)	110(11.80)		
Persistent	10a(4.46)	13a(4.51)	13a(5.06)	1a(1.56)	0a(0)	37(3.97)		
Intermittent	165a(73.66)	191a(66.32)	174a(67.70)	42a(65.63)	87b(87.88)	659(70.71)		
Never	31a, b(13.84)	57b(19.79)	22a, c(8.56)	13a, b(20.31)	3c(3.03)	126(13.53)		
Hearing impairment							33.943	0.001*
Had	18a(8.04)	24a(8.33)	52b(20.23)	5a, b(7.81)	17a, b(17.17)	116(12.45)		
Persistent	5a(2.23)	12a(4.17)	10a(3.89)	3a(4.69)	3a(3.03)	33(3.54)		
Intermittent	156a(69.64)	171a(59.38)	161a(62.65)	43a(67.19)	69a(69.7)	600(64.37)		
Never	45a, b(20.09)	81b(28.13)	34a(13.23)	13a, b(20.31)	10a(10.1)	183(19.64)		
Swallowing problems							43.9	0.000 *
Had	25a(11.16)	27a(9.38)	44a, b(17.12)	5a, b(7.81)	24b(24.24)	125(13.41)		
Persistent	6a(2.68)	7a(2.43)	17a(6.61)	2a(3.13)	3a(3.03)	19(2.04)		
Intermittent	161a(71.88)	194a, b(67.36)	171a, b(62.65)	43a, b(67.19)	55b(55.56)	614(66.95)		
Never	32a(14.29)	60a(20.83)	41a(13.62)	14a(21.88)	17a(17.17)	158(17.60)		
Olfactory impairment							33.943	0.001*
Had	28a, b(12.50)	28b(9.72)	49a(19.07)	8a, b(12.50)	10a, b(10.1)	123(13.20)		
Persistent	16a, b(7.14)	26b(9.03)	8a(3.11)	4a, b(6.25)	3a, b(3.03)	57(6.12)		
Intermittent	146a, b, c(65.18)	190a, b, c(65.97)	158c(61.48)	36a, c(56.25)	78b(78.79)	608(65.24)		
Never	34a(15.18)	44a(15.28)	42a(16.34)	16a(25.00)	8a(8.08)	144(15.44)		
Tactile impairment							43.742	0.000 *
Had	34a(15.18)	46a(15.97)	42a(16.34)	15a(23.44)	15a(15.15)	152(16.31)		
Persistent	5a(2.23)	14a, b(4.86)	21b(8.17)	5a, b(7.81)	2a, b(2.02)	47(5.04)		
Intermittent	138a(61.61)	180a(62.50)	166a(64.59)	41a, b(64.06)	80b(80.81)	605(64.91)		
Never	47a(20.98)	48a, b(16.67)	28b, c(10.89)	3b, c(4.69)	2c(2.02)	128(13.74)		
The amount of study and time							32.102	0.042*
None	167a(74.55)	207a(71.88)	203a(78.99)	46a(71.88)	85a(85.86)	708(75.97)		
Learning volume decreases for the same amount of time	39a(17.41)	40a(13.89)	36a(14.01)	8a(12.50)	9a(9.09)	132(14.16)		
The same amount of study and less time	15a(6.70)	27a(9.38)	16a(6.23)	7a(10.794)	4a(4.04)	69(7.40)		
No change	0a(0)	4a(1.39)	2a(0.78)	0a(0)	0a(0)	6(0.64)		
Both decrease	3a, b, c(1.34)	10c(3.47)	0b(0)	3a, c(4.69)	1a, b, c(1.01)	17(1.83)		
Fear of reinfection in a more serious form							27.267	0.000 *
No	99a, b(44.20)	138b, c(47.92)	85a(33.07)	31a, b, c(48.44)	61c(61.62)	414(44.42)		
Yes	125a, b(55.80)	150b, c(52.08)	172a(66.93)	33a, b, c(51.56)	38c(38.38)	518(55.58)		
Complications after developing a PASC							14.902	0.005*
No	105a, b(46.88)	156a, b(54.17)	110b(42.80)	34a, b(53.13)	62a(62.63)	467(50.11)		
Yes	119a, b(53.13)	132a, b(45.83)	147b(57.20)	30a, b(46.88)	37a(37.37)	465(49.89)		
Social isolation/decrease in social relationships/worsening of friendships							8.588	0.072
No	203a(90.63)	270a(93.75)	232a(90.27)	63a(98.44)	95a(95.96)	863(92.60)		
Yes	21a(9.38)	18a(6.25)	25a(9.73)	1a(1.56)	4a(4.04)	69(7.40)		
Family finances become difficult							6.522	0.163
No	180a(80.36)	241a(83.68)	202a(78.60)	56a(87.50)	87a(87.88)	766(82.19)		
Yes	44a(19.64)	47a(16.32)	55a(21.40)	8a(12.50)	12a(12.12)	166(17.81)		
Academic delays							8.385	0.078
No	172a(76.79)	230a(79.86)	186a(72.37)	55a(85.94)	81a(81.82)	724(77.68)		
Yes	52a(23.21)	58a(20.14)	71a(27.63)	9a(14.06)	18a(18.18)	208(22.32)		
Nothing to worry about							22.77	0.000 *
No	161a, b(71.88)	195b, c(67.71)	205a(79.77)	44a, b, c(68.75)	55c(55.56)	660(70.82)		
Yes	63a, b(28.13)	93b, c(32.29)	52a(20.23)	20a, b, c(31.25)	44c(44.44)	272(29.18)		

Note: + positive,—negative, each subscript letter indicates a subset of the class category, *P* < 0.05*, PASC Post-acute sequelae of SARS-CoV-2

### Analysis of score variability across different profiles

Table 4 presents the mean ± standard deviation of each key variable across the five latent profiles, along with ANOVA results and Bonferroni-corrected post-hoc differences. For PTSD symptoms, the order was Class 4 > Class 2 > Class 1 > Class 3 > Class 5. Conversely, for psychological resilience, the reverse order was observed: Class 5 > Class 4 > Class 3 > Class 2 > Class 1 (all Bonf‑*P* < 0.001). For well‑being (WHO‑5), Class 5 showed the highest scores, followed by Class 3, with Classes 1 and 2 showing intermediate levels and Class 4 the lowest. Full details of all pairwise comparisons, including subscale analyses, are provided in Supplementary Table S5.

**Table 4:** Score variability analysis of items in different profiles (*n* = 932)

Variables	Class 1	Class 2	Class 3	Class 4	Class 5	F	*P*	Post-hoc differences (Bonferroni-corrected)
**PCL-C**	37.72 ± 13.79	46.42** ± 12.0**	27.82 ± 11.23	47.68 ± 14.03	20.84 ± 7.19	139.649	0.000**	C4 > C2 > C1 > C3 > C5
Re-experiencing	11.67 ± 5.07	13.38 ± 5.049	8.25 ± 4.74	12.44 ± 5.35	6.41 ± 3.01	62.954	0.000**	C2 > C4 > C1 > C3 > C5
Avoidance	15.65 ± 6.59	18.84 ± 5.933	10.34 ± 4.73	19.02 ± 7.43	8.17 ± 2.45	120.877	0.000**	C4 > C2 > C1 > C3 > C5
Hypervigilance	10.40 ± 4.9	14.19 ± 4.726	9.23 ± 4.81	16.22 ± 5.19	6.25 ± 2.88	88.901	0.000**	C4 > C2 > C3 ≈ C1 > C5
**CD-RISC**	57.70 ± **17.16**	68.29 ± **15.56**	76.06 ± **11.52**	78.51 ± **19.24**	104.68 ± **14.61**	176.31	0.000**	C5 > **C4** > **C3** > **C2** > **C1**
Resilience	31.85 ± 11.85	33.94 ± 9.92	38.73 ± 7.46	39.44 ± 11.15	53.81 ± 8.56	98.378	0.000**	C5 > C4 > C3 > C2 > C1
Strength	17.655 ± 6.19	22.40 ± 5.70	25.07 ± 4.26	25.44 ± 6.90	33.88 ± 4.59	163.954	0.000**	C5 > *C4* > *C3* > *C2* > *C1*
Optimism	8.19 ± 4.18	11.95 ± 3.73	12.27 ± 3.04	13.62 ± 4.55	16.99 ± 2.74	110.564	0.000**	C5 > C4 > C3 > C2 > C1
**WHO-5**	11.98 ± 5.63	12.22 ± 5.72	14.81 ± 6.38	11.62 ± 5.62	19.64 ± 8.30	34.932	0.000**	C5 > C3 > C2 ≈ C1 > C4
**Negative emotion**	22.46 ± **9.17**	23.29 ± **9.33**	24.20 ± **8.51**	25.78 ± **9.56**	18.48 ± **8.23**	9.230	0.000**	C4 > C3 ≈ C2 ≈ C1 > C5
Negative emotion of lifestyles	11.25 ± 5.3	11.44 ± 5.32	11.91 ± 4.78	11.75 ± 5.27	9.42 ± 4.35	115.321	0.001**	C3 > C4 ≈ C2 ≈ C1 > C5
Negative emotion of learning	9.19 ± 4.49	9.66 ± 4.470	9.93 ± 4.19	11.54 ± 5.12	7.24 ± 3.39	210.462	0.000**	C4 > C3 ≈ C2 ≈ C1 > C5

Data are presented as Mean ± Standard Deviation

*PCL-C* Posttraumatic Stress Disorder Checklist-Civilian Version, *CD-RISC* Connor-Davidson Resilience Scale, *WHO-5* World Health Organization Five-item Well-Being Index

**P < 0.001; *P < 0.05. Significant Post-Hoc Ordering: This column presents the statistically significant hierarchical relationships among profiles (C1-C5) based on Bonferroni-corrected pairwise comparisons (see Supplementary Table S3 for full details). The “ > ” symbol indicates a statistically significant difference (Bonf-*p* < 0.05). Groups connected by ≈ (e.g., “C4 ≈ C2”) are not significantly different from each other. Only significant differences are depicted. Unbolded terms represent sub-dimensions of the corresponding scale

### Multinomial logistic regression analysis of depression profiles

The study first screened for statistically significant variables through univariate analysis to serve as candidate independent variables. To prevent multicollinearity from interfering with the regression results, variance inflation factor (VIF) diagnostics were performed on the candidate variables. The results indicated severe multicollinearity (VIF > 10) for several variables, including sleep problems (VIF = 10.312), difficulty concentrating (VIF = 11.033), and hearing problems (VIF = 11.944), which were subsequently removed. After controlling for potential confounding factors such as gender, age, grade, and BMI, the final multivariable multinomial logistic regression model, with the "high resilience-low PTSD" class (Class 5) as the reference group, is presented in Table 5.

**Table 5:** The multifactor analysis of PCL -C and CD-RISC of adolescents by multi-logistic regression (*n* = 932)

Variable	Class 1 VS Class 5				Class 2 VS Class 5				Class 3 VS Class 5				Class 4 VS Class 5			
	β	OR	95%CI	*P*	β	OR	95%CI	*P*	β	OR	95%CI	*P*	β	OR	95%CI	*P*
Gender(ref: male)	−0.203	0.816	(0.465,1.431)	0.478	−0.158	0.854	(0.492,1.483)	0.576	0.426	1.53	(0.863,2.713)	0.145	1.033	2.811	(1.376,5.743)	**0.005**
Number of vaccinations	−0.001	0.999	(0.891,1.121)	0.993	−0.061	0.941	(0.842,1.053)	0.288	0.178	1.194	(1.057,1.350)	0.004	0.346	1.414	(1.190,1.680)	**0.000**
Fatigue (ref: Never)	0.320	1.377	(0.642,2.952)	0.412	0.349	1.417	(0.673,2.984)	0.358	0.619	1.858	(0.837,4.123)	0.128	0.229	1.257	(0.485,3.255)	0.638
Weakness (ref: Never)	−0.167	0.846	(0.233,3.075)	0.799	−0.296	0.744	(0.211,2.621)	0.645	0.347	1.415	(0.371,5.394)	0.611	1.113	3.044	(0.491,18.879)	0.232
Memory loss(ref: Never)	0.983	2.672	(0.622,11.482)	0.186	0.498	1.646	(0.404,6.710)	0.487	0.155	1.168	(0.257,5.303)	0.841	−0.307	0.736	(0.098,5.538)	0.766
Constipation/diarrhoea (ref: Never)	−0.222	0.801	(0.258,2.483)	0.700	0.255	1.29	(0.425,3.918)	0.653	0.774	2.167	(0.627,7.493)	0.222	0.344	1.41	(0.302,6.582)	0.662
Anxiety/depression (ref: Never)	−0.706	0.494	(0.159,1.531)	0.221	−0.666	0.514	(0.170,1.556)	0.239	−0.820	0.440	(0.137,1.412)	0.168	−0.312	0.732	(0.177,3.028)	0.666
Dizziness (ref: Never)	−0.209	0.812	(0.259,2.544)	0.720	−0.505	0.604	(0.200,1.822)	0.371	−0.012	0.988	(0.307,3.180)	0.984	−0.269	0.764	(0.193,3.026)	0.701
Slow movement (ref: Never)	0.105	1.111	(0.366,3.374)	0.853	−0.051	0.95	(0.324,2.784)	0.926	−0.479	0.620	(0.202,1.904)	0.403	−0.067	0.935	(0.230,3.807)	0.926
Swallowing problems (ref: Never)	0.306	1.358	(0.453,4.069)	0.585	0.264	1.302	(0.446,3.804)	0.629	0.058	1.060	(0.347,3.234)	0.918	0.293	1.341	(0.324,5.553)	0.686
Olfactory impairment (ref: Never)	0.031	1.031	(0.255,4.171)	0.965	0.600	1.823	(0.457,7.268)	0.395	0.336	1.399	(0.326,6.012)	0.652	0.841	2.320	(0.314,17.123)	0.409
Tactile impairment (ref: Never)	0.117	1.124	(0.329,3.843)	0.852	−0.199	0.82	(0.243,2.768)	0.749	−0.078	0.925	(0.256,3.350)	0.906	−0.225	0.799	(0.137,4.661)	0.803
Neither study nor times no change. (ref: None)	−0.290	0.748	(0.307,1.824)	0.523	−0.595	0.552	(0.227,1.340)	0.189	−0.323	0.724	(0.293,1.791)	0.485	−0.264	0.768	(0.241,2.452)	0.656
Both study and times decrease. (ref: None)	0.038	1.039	(0.429,2.519)	0.932	0.121	1.129	(0.477,2.672)	0.783	0.084	1.088	(0.446,2.656)	0.854	−0.030	0.971	(0.321,2.932)	0.958
Fear of reinfection in a more serious form (ref: yes)	−0.664	0.515	(0.259,1.022)	0.058	−0.771	0.463	(0.235,0.909)	**0.025**	−0.987	0.373	(0.189,0.737)	**0.005**	−0.386	0.680	(0.308,1.502)	0.340
Complications after developing a PASC(ref: yes)	0.203	1.225	(0.631,2.379)	0.549	0.141	1.151	(0.600,2.210)	0.672	−0.017	0.983	(0.509,1.898)	0.959	0.127	1.136	(0.527,2.448)	0.745
Nothing to worry about(ref: yes)	0.446	1.561	(0.868,2.808)	0.137	0.058	1.059	(0.599,1.873)	0.843	0.680	1.974	(1.090,3.576)	**0.025**	0.028	1.028	(0.525,2.015)	0.936

Note: Class 1 low resilience-medium PTSD, Class 2 moderately low resilience-moderately high PTSD, Class 3 medium resilience-moderately low PTSD, Class 4 moderately high resilience-high PTSD, Class 5 high resilience-low PTSD, OR oddratio,95%CI Confidence Interval, bold *P* < 0.05, WHO-5 World Health Organization Five-item Well-Being Index, PASC Post-acute sequelae of SARS-CoV-2

The analysis revealed several significant predictors of latent class membership. Women was associated with 2.81 times higher odds (95% CI: 1.38–5.74) of belonging to the "moderately high resilience-high PTSD" class (Class 4) compared to the reference class (Class 5). A higher number of COVID-19 vaccinations was a significant predictor for membership in both the "medium resilience-moderately low PTSD" class (Class 3: OR = 1.19, 95% CI: 1.06–1.35, *P* = 0.004) and Class 4 (OR = 1.41, 95% CI: 1.19–1.68, *P* < 0.001). Psychological factors related to the infection experience were also significant. Fear of reinfection in a more severe form served as a protective factor, significantly reducing the odds of belonging to the "moderately low resilience-moderately high PTSD" class (Class 2: OR = 0.46, 95% CI: 0.24–0.91, *P* = 0.025) and Class 3 (OR = 0.37, 95% CI: 0.19–0.74, *P* = 0.005) compared to Class 5. Conversely, holding the attitude that there was "nothing to worry about" was associated with 1.97 times higher odds (95% CI: 1.09–3.58, *P* = 0.025) of belonging to Class 3. Other long COVID physical symptoms, changes in learning patterns, and most post-recovery concerns were not statistically significant predictors in the final multivariable model.

## Discussion

Employing latent profile analysis, this study is the first to delineate the heterogeneous co-occurrence patterns of PTSD and the psychological resilience among Chinese adolescents experiencing Long COVID. The central finding is the identification of five distinct psychological adaptation profiles, ranging from "high resilience-low PTSD" to "low resilience-moderate PTSD". This result fundamentally confirms that adolescents' psychological responses to Long COVID as a chronic stressor are not homogeneous but exhibit qualitative differences. Of particular significance is that over half of the adolescents (58.5%) were distributed across two intermediate profiles: "moderately low resilience-moderately high PTSD" (30.93%) and "medium resilience-moderately low PTSD" (27.61%). This suggests that the majority of adolescents exist on an intermediate continuum, where their adaptive state is characterized neither by severe dysfunction nor by complete health, but rather by varying equilibrium points between symptom burden and resilience resources. The most theoretically provocative finding is the existence of the "moderately high resilience-high PTSD" profile, indicating that a high level of psychological resilience does not necessarily preclude significant distress; the two can coexist, forming a distinct adaptive state. This distribution highlights that most individuals reside on a continuum of adaptation, complementing previous research that treated PTSD symptoms or resilience as independent variables and balancing varying degrees of symptom burden and resilience resources, which has direct implications for tiered public health intervention.

Class 2 ("moderately low resilience-moderately high PTSD", 30.93%), as the single largest profile, is characterized by significant PTSD symptoms (particularly avoidance and hyperarousal) coexisting with relatively insufficient resilience resources. This strongly supports the view of the pandemic as a chronic stressor leading to the depletion of psychological resources [31]. This group likely represents individuals who were vulnerable during the acute phase and whose coping resources have not been effectively replenished under sustained pressure. They align with the notion of 'symptomatic resilience' profile [43], maintaining basic function while enduring persistent distress. From a preventive standpoint, "moderately low resilience-moderately high PTSD" profile constitutes a critical intervention window. Targeted resilience-building programs (e.g., focusing on cognitive flexibility, emotion regulation) are indicated as a priority over direct trauma-focused therapy, aiming to prevent deterioration towards the more impaired "low resilience-medium PTSD" profile state.

In contrast, class 3 ("medium resilience-moderately low PTSD", 27.61%)), presents a more adaptive equilibrium. This group exhibits milder symptoms and possesses a moderate level of resilience, potentially positioned on a more stable or recovery-oriented trajectory, possibly benefiting from personal strength, optimistic tendencies, or effective social support [25]. However, their medium level of resilience implies a degree of vulnerability to future stressors. The focus for this group should be consolidation and enhancement, reinforcing their existing resilience level through mental health education and peer support programs to encourage progression towards the "high resilience-low PTSD" profile profile.

Class 4 ("moderately high resilience-high PTSD", 6.77%) constitutes the theoretical keystone of this study. The existence of this profile suggests that psychological resilience is a multidimensional construct. The high CD-RISC scores in "moderately high resilience-high PTSD" profile may predominantly reflect dimensions like perseverance and optimism, enabling goal-directed behavior amidst high distress. It potentially supported by robust social networks [14] or learned adaptive coping [17]. Concurrently, difficulties in other resilience dimensions, such as emotional regulation or trauma integration, may underlie the persistence of severe PTSD symptoms. This profile resonates with findings of 'struggling yet resilient' individuals in chronic illness populations and necessitates future research examining the differential role of resilience subdimensions.

Our five-profile structure differs from the three or four profiles commonly reported in studies of adolescents during the acute pandemic phase [53]. This discrepancy likely stems from our focus on the specific, persistent stressor of Long COVID and the simultaneous integration of resilience as a core variable. The overall prevalence of significant PTSD symptoms aligns with documented pandemic-related trauma in youth [33], while the identification of the optimal "high resilience-low PTSD" profile corroborates research on positive adaptation [43]. The finding that women were more likely to belong to "moderately high resilience-high PTSD" profile continues the discourse on gender differences in trauma response [12], possibly reflecting distinct patterns in emotional processing and social support roles among women. One of the most practically significant findings is that a higher number of vaccine doses was associated with more adaptive profiles ("medium resilience-moderately low PTSD" profile, "moderately high resilience-high PTSD" profile). This suggests vaccination may confer psychological empowerment by reducing fear of reinfection and enhancing perceived control, thereby contributing to resilience [20, 49].

To integrate these findings, we propose a preliminary 'Differential Susceptibility–Dynamic Adaptation' framework. First, an individual's innate differential susceptibility (e.g., biological traits sensitive to stress) forms the baseline for the breadth and depth of their stress response to Long COVID. Building upon this, psychological adaptation is a dynamic systems process [27], where subsystems such as emotion, cognition, and social behavior interact nonlinearly under pressure, eventually self-organizing into relatively stable "attractor states"—namely, the five profiles revealed in this study. "moderately high resilience-high PTSD" profile can be understood as a specific 'metastable attractor' arising under conditions of high susceptibility and sustained pressure. Environmental factors (e.g., vaccination, social support) then act as key parameters, modulating the system's trajectory towards healthier or more pathological attractors. This framework elevates the profile findings of this study from static classification to an understanding of dynamic adaptive processes, providing theoretical guidance for future exploration of profile stability, predictors of transition, and timing for personalized intervention.

The findings directly support a tiered and precision-oriented mental health service system. For adolescents with "low resilience" (Classes 1 and 2), interventions should prioritize resilience-building. Considering that adolescents often rely on support from loved ones [4], relevant interventions must be firmly grounded in family support as a crucial pillar, focusing on using emotional companionship and positive guidance from family members to help adolescents enhance coping skills, foster optimism, and further strengthen their perceived social support [8]. For "moderately low resilience-moderately high PTSD" profile, universal school-based resilience enhancement programs should be implemented. For "medium resilience-moderately low PTSD" profile, consolidative group counseling and peer support should be conducted, encouraging them to share adaptive strategies through workshops or peer leadership programs, transforming their experiences into resources for helping others while further reinforcing their own psychological capital. For "moderately high resilience-high PTSD" profile, their existing resilience can be leveraged as a therapeutic asset, but they require direct, trauma-focused interventions (e.g., trauma-focused cognitive behavioral therapy) to address their pronounced PTSD symptoms. "high resilience-low PTSD" profile can serve as a potential resource for peer support programs, modeling adaptive strategies for others.

The finding linking vaccination to more adaptive psychological profiles provides strong and novel information for public health campaigns. Promoting vaccination can serve not only as a physical health measure but also as a step towards restoring psychological safety and control, thereby reducing pandemic-related anxiety and trauma. Policymakers and educational institutions should acknowledge the enduring psychological impact of the pandemic. Incorporating mental health literacy and resilience training into school curricula, ensuring accessible counseling services, and promoting vaccination are evidence-based strategies supported by this study.

## Limitations

This study has some shortcomings that should be considered and improved in future research. (1) The cross-sectional design precludes the examination of temporal relationships and causal inferences. We cannot determine whether the identified latent profiles are stable over time or how symptoms and resilience influence each other longitudinally. Additionally, data collection took place between March and May 2023, a considerable time after the 2th peak of the COVID-19 pandemic in China. This distance may influence the recall and reporting of pandemic-related traumatic experiences and symptoms, potentially affecting the accuracy and ecological validity of the measured psychological responses. To address these gaps, future research could adopt a mixed-methods design: first identifying distinct latent profiles through quantitative latent profile analysis, then recruiting participants from each profile to conduct in-depth qualitative interviews. This integrated approach would help unpack the detailed contextual factors, personal experiences, and underlying mechanisms shaping the coexistence patterns of PTSD and resilience, thereby complementing the limitations of a purely quantitative analysis and yielding more comprehensive insights. (2) Recruitment from a single vocational college limits generalizability. Findings may not extend to adolescents in other educational or regional contexts, or other regions with different pandemic policies and healthcare resources, or younger age groups. Multi-center studies with diverse samples are required to validate and generalize the profiles. (3) Self-report measures are susceptible to recall and social desirability biases, particularly for past traumatic events assessed months after the pandemic peak. This may affect the accuracy of symptom reporting. The "COVID-19 negative emotion situation scale," while developed with expert input, is a newly adapted measure requiring further validation in diverse populations. Future research should incorporate clinician-administered or time-proximal assessments to enhance validity. (4) Despite employing latent profile analysis to explore heterogeneity, the profiles are data-driven and require replication in independent samples. Although we controlled for several demographic variables in the logistic regression, the potential for residual confounding by unmeasured factors (e.g., socioeconomic status, pre-pandemic mental health, family dynamics) remains. External validation in subsequent cohorts is essential.

## Conclusion

Employing Latent Profile Analysis (LPA), this study identified five distinct co-occurring patterns of post-traumatic stress disorder (PTSD) symptoms and psychological resilience among adolescents experiencing Long COVID, unveiling considerable heterogeneity in their psychological sequelae. The central finding indicates that psychosocial and behavioral factors, such as fear of reinfection, vaccination status, and gender, play a more pivotal role in differentiating adaptive outcomes than the array of non-specific physical symptoms examined. Notably, a substantial proportion of adolescents were classified into intermediate profiles, including "moderately low resilience–moderately high PTSD" profile and "medium resilience-moderately low PTSD" profile, constituting a priority at-risk subgroup requiring proactive attention. Based on these findings, 1) For the largest at-risk subgroup, universal school-based resilience-building programs should be implemented, focusing particularly on enhancing the Strength and Optimism dimensions of resilience; 2) For those demonstrating moderate recovery, targeted supportive interventions should be provided to consolidate adaptive gains and prevent relapse; 3) For individuals with pronounced symptomatology, access to specialized, trauma-focused therapy must be ensured. It would be valuable for future research to explore additional mediating or moderating variables, including but not limited to social support networks and specific coping strategies, to further innovate the conceptual framework.

## Supplementary Information

Supplementary Material 1.

Supplementary Material 2.

Supplementary Material 3.

Supplementary Material 4.

Supplementary Material 5.

Supplementary Material 6.

## Data Availability

The datasets used and/or analysed during the current study are available from the corresponding author on reasonable request.
