# Supplementary figures and images for: Latent profile analysis of the symptoms for posttraumatic stress disorder and psychological resilience in Chinese adolescents experiencing post Covid-19: a quantetative study
Source: BMC Psychol. 2026 Apr 7;14:712. doi: 10.1186/s40359-026-03987-8 (PMC13173930; doi:10.1186/s40359-026-03987-8)

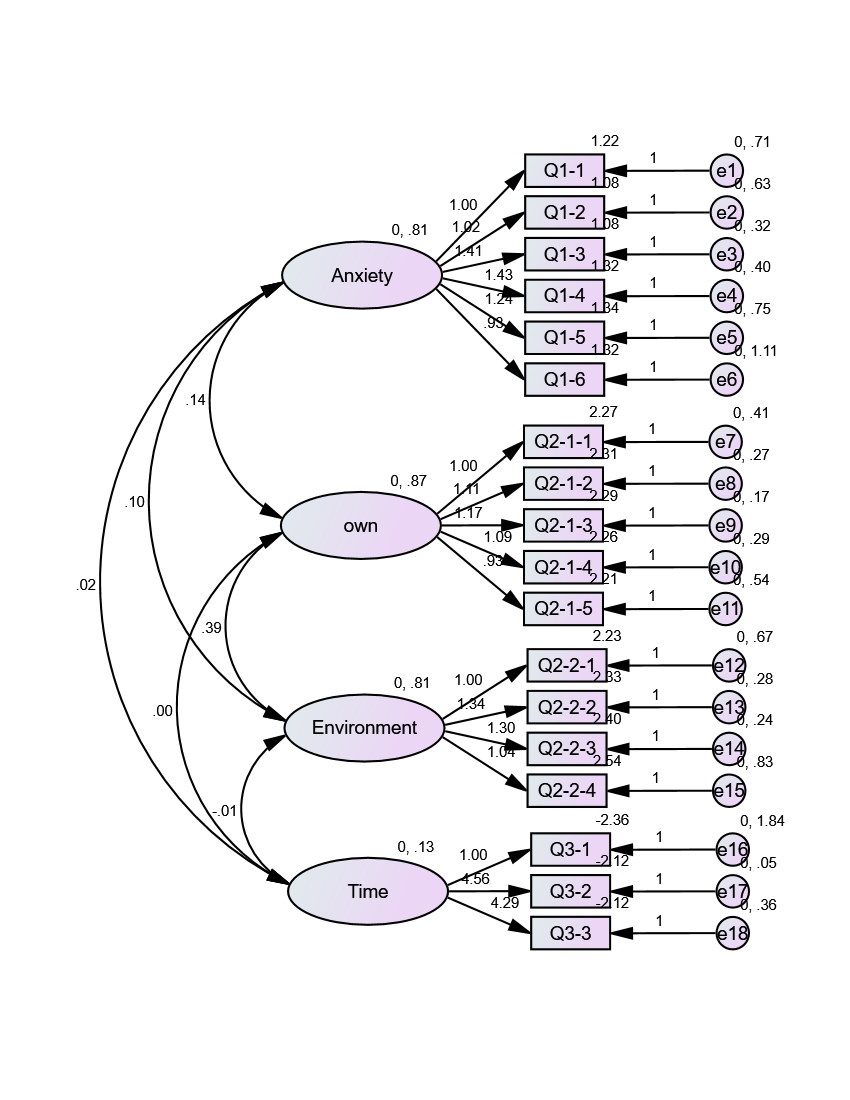


Supplementary Fig S1. final model of CFA.

Supplement: Supplementary file 1 — Supplementary Material 1. [file 40359_2026_3987_MOESM1_ESM.docx]
