# Supplementary material for: Latent profile analysis of the symptoms for posttraumatic stress disorder and psychological resilience in Chinese adolescents experiencing post Covid-19: a quantetative study
Source: BMC Psychol. 2026 Apr 7;14:712. doi: 10.1186/s40359-026-03987-8 (PMC13173930; doi:10.1186/s40359-026-03987-8)
Supplement: Supplementary file 2 — Supplementary Material 2. [file 40359_2026_3987_MOESM2_ESM.docx]

| Supplementary Table S1. consistency reliability and convergent validity of the academic impact scale. | | | | | | | | | | |
| --- | --- | --- | --- | --- | --- | --- | --- | --- | --- | --- |
| Subdimension | |  |  |  | Sat-Estimate | S.E. | *P* | AVE | CR | Cronbach's α |
| Anxiety | | Q1-1 | <--- | Anxiety | 0.804 |  |  | 0.673 | 0.924 | 0.904 |
|  |  | Q1-2 | <--- | Anxiety | 0.825 | 0.044 | *** |  |  |  |
|  |  | Q1-3 | <--- | Anxiety | 0.922 | 0.050 | *** |  |  |  |
|  |  | Q1-4 | <--- | Anxiety | 0.891 | 0.052 | *** |  |  |  |
|  |  | Q1-5 | <--- | Anxiety | 0.810 | 0.052 | *** |  |  |  |
|  |  | Q1-6 | <--- | Anxiety | 0.639 | 0.050 | *** |  |  |  |
| Negative emotion | Lifestyles | Q2-1-1 | <--- | Negative emotion_lifestyles | 0.849 |  |  | 0.746 | 0.936 | 0.935 |
|  |  | Q2-1-2 | <--- | Negative emotion_lifestyles | 0.900 | 0.032 | *** |  |  |  |
|  |  | Q2-1-3 | <--- | Negative emotion_lifestyles | 0.913 | 0.031 | *** |  |  |  |
|  |  | Q2-1-4 | <--- | Negative emotion_lifestyles | 0.876 | 0.032 | *** |  |  |  |
|  |  | Q2-1-5 | <--- | Negative emotion_lifestyles | 0.773 | 0.034 | *** |  |  |  |
|  | Learning | Q2-2-1 | <--- | Negative emotion_learning | 0.705 |  |  | 0.689 | 0.898 | 0.890 |
|  |  | Q2-2-2 | <--- | Negative emotion_learning | 0.904 | 0.047 | *** |  |  |  |
|  |  | Q2-2-3 | <--- | Negative emotion_learning | 0.900 | 0.045 | *** |  |  |  |
|  |  | Q2-2-4 | <--- | Negative emotion_learning | 0.796 | 0.047 | *** |  |  |  |
| Time and amount of study | | Q3-1 | <--- | Time and amount of study | 0.965 |  |  | 0.636 | 0.812 | 0.864 |
|  |  | Q3-2 | <--- | Time and amount of study | 0.990 | 0.611 | *** |  |  |  |
|  |  | Q3-3 | <--- | Time and amount of study | 0.930 | 0.547 | *** |  |  |  |
| Note:***: *P* ＜0.001; AVE Average Variance Extracted; CR Construct Reliability. | | | | | | | | | | |
