# Supplementary material for: Latent profile analysis of the symptoms for posttraumatic stress disorder and psychological resilience in Chinese adolescents experiencing post Covid-19: a quantetative study
Source: BMC Psychol. 2026 Apr 7;14:712. doi: 10.1186/s40359-026-03987-8 (PMC13173930; doi:10.1186/s40359-026-03987-8)
Supplement: Supplementary file 3 — Supplementary Material 3. [file 40359_2026_3987_MOESM3_ESM.docx]

| Supplementary Table S2. The model fit indices before and after adding the CLF | | | | | | | |
| --- | --- | --- | --- | --- | --- | --- | --- |
| **Model Type** | **χ²** | **df** | **χ²/df** | **CFI** | **RMSEA** | **CMB Variance Explained** | **Max Loading Change** |
| Original CFA Model | 2806.676 | 222 | 13.692 | 0.879 | 0.003 | - | - |
| CFA + Common Latent Factor (CLF) | 2703.629 | 258 | 10.479 | 0.892 | 0.002 | 0.327 | 0.12 |
| Chi-square Difference | 43.047 | 36 | - | - | - | - | - |
| p-value (Δχ²) | 0.195 | - | - | - | - | - | - |
| Threshold | - | - | < 5 | ≥ 0.8 | ≤ 0.08 | ≤ 40% | ≤ 0.20 |
