# Supplementary material for: Latent profile analysis of the symptoms for posttraumatic stress disorder and psychological resilience in Chinese adolescents experiencing post Covid-19: a quantetative study
Source: BMC Psychol. 2026 Apr 7;14:712. doi: 10.1186/s40359-026-03987-8 (PMC13173930; doi:10.1186/s40359-026-03987-8)
Supplement: Supplementary file 4 — Supplementary Material 4. [file 40359_2026_3987_MOESM4_ESM.docx]

| Supplementary Table S3. Common method bias test using the common latent factor (CLF) method. | | | | | |
| --- | --- | --- | --- | --- | --- |
| **Construct** | **Indicator** | **Substantive Factor Loading (R1)** | **R1²** | **Method Factor Loading (R2)** | **R2²** |
| **COVID-19 Negative Emotion Scale** | | | | | |
| Anxiety Situation | NE1 | 0.897*** | 0.804 | 0.011+ | 0.0001 |
|  | NE2 | 0.912*** | 0.832 | 0.009 | 0.0001 |
|  | NE3 | 0.876*** | 0.767 | 0.013+ | 0.0002 |
|  | NE4 | 0.903*** | 0.815 | 0.008 | 0.0001 |
|  | NE5 | 0.889*** | 0.79 | 0.010+ | 0.0001 |
| Negative Impact on Learning (Personal) | NE6 | 0.864*** | 0.746 | 0.012+ | 0.0001 |
|  | NE7 | 0.891*** | 0.794 | 0.007 | 0.0001 |
|  | NE8 | 0.858*** | 0.736 | 0.014+ | 0.0002 |
|  | NE9 | 0.915*** | 0.837 | 0.006 | 0.0001 |
| Negative Impact on Learning (Environmental) | NE10 | 0.872*** | 0.76 | 0.011+ | 0.0001 |
|  | NE11 | 0.886*** | 0.785 | 0.009 | 0.0001 |
|  | NE12 | 0.869*** | 0.755 | 0.013+ | 0.0002 |
| Learning Time & Amount | NE13 | 0.853*** | 0.728 | 0.015+ | 0.0002 |
|  | NE14 | 0.895*** | 0.801 | 0.008 | 0.0001 |
|  | NE15 | 0.878*** | 0.771 | 0.010+ | 0.0001 |
|  | NE16 | 0.861*** | 0.742 | 0.012+ | 0.0001 |
|  | NE17 | 0.883*** | 0.78 | 0.009 | 0.0001 |
|  | NE18 | 0.907*** | 0.822 | 0.007 | 0.0001 |
| **WHO-5 Well-Being Index** | | | | | |
| Well-Being | WHO5-1 | 0.864*** | 0.746 | 0.006 | 0.0001 |
|  | WHO5-2 | 0.891*** | 0.794 | 0.009 | 0.0001 |
|  | WHO5-3 | 0.905*** | 0.819 | 0.005 | 0.0001 |
|  | WHO5-4 | 0.879*** | 0.773 | 0.008 | 0.0001 |
|  | WHO5-5 | 0.887*** | 0.787 | 0.007 | 0.0001 |
| **PCL-C** | | | | | |
| Re-experiencing | PCL1 | 0.873*** | 0.762 | 0.008 | 0.0001 |
|  | PCL2 | 0.856*** | 0.733 | 0.013+ | 0.0002 |
|  | PCL3 | 0.892*** | 0.796 | 0.009 | 0.0001 |
|  | PCL4 | 0.868*** | 0.753 | 0.011+ | 0.0001 |
|  | PCL5 | 0.884*** | 0.781 | 0.010+ | 0.0001 |
| Avoidance | PCL6 | 0.859*** | 0.738 | 0.012+ | 0.0001 |
|  | PCL7 | 0.871*** | 0.759 | 0.010+ | 0.0001 |
|  | PCL8 | 0.893*** | 0.797 | 0.008 | 0.0001 |
|  | PCL9 | 0.865*** | 0.748 | 0.011+ | 0.0001 |
|  | PCL10 | 0.886*** | 0.785 | 0.009 | 0.0001 |
|  | PCL11 | 0.879*** | 0.773 | 0.010+ | 0.0001 |
|  | PCL12 | 0.895*** | 0.801 | 0.007 | 0.0001 |
| Hypervigilance | PCL13 | 0.862*** | 0.743 | 0.013+ | 0.0002 |
|  | PCL14 | 0.881*** | 0.776 | 0.010+ | 0.0001 |
|  | PCL15 | 0.897*** | 0.804 | 0.008 | 0.0001 |
|  | PCL16 | 0.874*** | 0.764 | 0.011+ | 0.0001 |
|  | PCL17 | 0.889*** | 0.79 | 0.009 | 0.0001 |
| **CD-RISC** | | | | | |
| Resilience | CD11 | 0.902*** | 0.814 | 0.007 | 0.0001 |
|  | CD12 | 0.886*** | 0.785 | 0.009 | 0.0001 |
|  | CD13 | 0.893*** | 0.797 | 0.008 | 0.0001 |
|  | CD14 | 0.911*** | 0.83 | 0.006 | 0.0001 |
|  | CD15 | 0.878*** | 0.771 | 0.010+ | 0.0001 |
|  | CD16 | 0.869*** | 0.755 | 0.012+ | 0.0001 |
|  | CD17 | 0.883*** | 0.78 | 0.009 | 0.0001 |
|  | CD18 | 0.895*** | 0.801 | 0.007 | 0.0001 |
|  | CD19 | 0.872*** | 0.76 | 0.011+ | 0.0001 |
|  | CD20 | 0.889*** | 0.79 | 0.010+ | 0.0001 |
|  | CD21 | 0.897*** | 0.804 | 0.008 | 0.0001 |
|  | CD22 | 0.905*** | 0.819 | 0.006 | 0.0001 |
|  | CD23 | 0.881*** | 0.776 | 0.010+ | 0.0001 |
| Strength | CD1 | 0.875*** | 0.766 | 0.012+ | 0.0001 |
|  | CD5 | 0.892*** | 0.796 | 0.009 | 0.0001 |
|  | CD7 | 0.868*** | 0.753 | 0.011+ | 0.0001 |
|  | CD8 | 0.884*** | 0.781 | 0.010+ | 0.0001 |
|  | CD9 | 0.879*** | 0.773 | 0.010+ | 0.0001 |
|  | CD10 | 0.891*** | 0.794 | 0.008 | 0.0001 |
|  | CD24 | 0.887*** | 0.787 | 0.009 | 0.0001 |
|  | CD25 | 0.895*** | 0.801 | 0.007 | 0.0001 |
| Optimism | CD2 | 0.864*** | 0.746 | 0.013+ | 0.0002 |
|  | CD3 | 0.871*** | 0.759 | 0.011+ | 0.0001 |
|  | CD4 | 0.883*** | 0.78 | 0.010+ | 0.0001 |
|  | CD6 | 0.876*** | 0.767 | 0.012+ | 0.0001 |
| **Average (by absolute value)** | - | - | 0.805 | - | 0.016 |
| Note: ***p<0.001, **p<0.01, *p<0.05, +=p<0.1; R1 = Standardized factor loading of items on substantive constructs; R1² = Variance explained by substantive constructs; R2 = Standardized factor loading of items on the common latent factor; R2² = Variance explained by the common latent factor; | | | | | |
