# Supplementary material for: Latent profile analysis of the symptoms for posttraumatic stress disorder and psychological resilience in Chinese adolescents experiencing post Covid-19: a quantetative study
Source: BMC Psychol. 2026 Apr 7;14:712. doi: 10.1186/s40359-026-03987-8 (PMC13173930; doi:10.1186/s40359-026-03987-8)
Supplement: Supplementary file 5 — Supplementary Material 5. [file 40359_2026_3987_MOESM5_ESM.docx]

| Supplementary Table S4. Average probability of attribution for each potential profile. | | | | | |
| --- | --- | --- | --- | --- | --- |
| **Class** | **Profile1** | **Profile2** | **Profile3** | **Profile4** | **Profile5** |
| Profile1 | 0.935 | 0.034 | 0.031 | 0.000 | 0.000 |
| Profile2 | 0.021 | 0.914 | 0.038 | 0.001 | 0.027 |
| Profile3 | 0.015 | 0.053 | 0.898 | 0.032 | 0.002 |
| Profile4 | 0.000 | 0.001 | 0.080 | 0.918 | 0.002 |
| Profile5 | 0.000 | 0.08 | 0.009 | 0.008 | 0.903 |
