# Supplementary material for: Latent profile analysis of the symptoms for posttraumatic stress disorder and psychological resilience in Chinese adolescents experiencing post Covid-19: a quantetative study
Source: BMC Psychol. 2026 Apr 7;14:712. doi: 10.1186/s40359-026-03987-8 (PMC13173930; doi:10.1186/s40359-026-03987-8)
Supplement: Supplementary file 6 — Supplementary Material 6. [file 40359_2026_3987_MOESM6_ESM.docx]

| Supplementary Table S5. Results of bonferroni-corrected post-hoc pairwise comparisons. | | | | | | | |
| --- | --- | --- | --- | --- | --- | --- | --- |
| **Scale** | **Class (i vs j)** | **Mean Δ (i–j)** | **SE** | **95% CI** | **Raw *P*** | **Bonf-*P*** | **Significance after Bonferroni correction** |
| WHO-5 | 1 vs 2 | –0.24 | 0.552 | (–1.79, 1.32) | 1 | 1 | ns |
|  | 1 vs 3 | –2.83 | 0.567 | (–4.43, 1.24) | <.001 | 0.003 | ** |
|  | 1 vs 4 | –7.66 | 0.748 | (–9.76, 5.55) | <.001 | <.001 | ** |
|  | 1 vs 5 | 0.36 | 0.884 | (–2.13, 2.85) | 1 | 1 | ns |
|  | 2 vs 3 | –2.59 | 0.532 | (–4.09, 1.10) | <.001 | 0.004 | ** |
|  | 2 vs 4 | –7.42 | 0.722 | (–9.45, 5.39) | <.001 | <.001 | ** |
|  | 2 vs 5 | 0.6 | 0.862 | (–1.83, 3.02) | 1 | 1 | ns |
|  | 3 vs 5 | 3.19 | 0.872 | (0.74, 5.64) | 0.001 | 0.01 | * |
|  | 4 vs 5 | 8.02 | 0.999 | (5.21, 10.83) | <.001 | <.001 | ** |
| PCL-C | 1 vs 2 | –8.70 | 1.068 | (–11.71, 5.70) | <.001 | <.001 | ** |
|  | 1 vs 3 | 9.9 | 1.095 | (6.82, 12.98) | <.001 | <.001 | ** |
|  | 1 vs 4 | 16.88 | 1.446 | (12.81, 20.95) | <.001 | <.001 | ** |
|  | 1 vs 5 | –9.96 | 1.709 | (–14.77, 5.16) | <.001 | <.001 | ** |
|  | 2 vs 3 | 18.6 | 1.028 | (15.71, 21.50) | <.001 | <.001 | ** |
|  | 2 vs 4 | 25.58 | 1.396 | (21.65, 29.51) | <.001 | <.001 | ** |
|  | 2 vs 5 | –1.26 | 1.667 | (–5.95, 3.43) | 1 | 1 | ns |
|  | 3 vs 4 | 6.98 | 1.417 | (2.99, 10.97) | <.001 | <.001 | ** |
|  | 3 vs 5 | –19.87 | 1.685 | (–24.61, 15.13) | <.001 | <.001 | ** |
|  | 4 vs 5 | –26.84 | 1.931 | (–32.28, 21.41) | <.001 | <.001 | ** |
| CD-RISC | 1 vs 2 | –10.60 | 1.354 | (–14.41, 6.79) | <.001 | <.001 | ** |
|  | 1 vs 3 | –18.37 | 1.389 | (–22.28, 14.46) | <.001 | <.001 | ** |
|  | 1 vs 4 | –46.98 | 1.833 | (–52.14, 41.83) | <.001 | <.001 | ** |
|  | 1 vs 5 | –20.82 | 2.165 | (–26.91, 14.73) | <.001 | <.001 | ** |
|  | 2 vs 3 | –7.77 | 1.302 | (–11.43, 4.11) | <.001 | <.001 | ** |
|  | 2 vs 4 | –36.39 | 1.768 | (–41.36, 31.41) | <.001 | <.001 | ** |
|  | 2 vs 5 | –10.22 | 2.111 | (–16.16, 4.28) | <.001 | 0.002 | ** |
|  | 3 vs 4 | –28.61 | 1.795 | (–33.67, 23.56) | <.001 | <.001 | ** |
|  | 3 vs 5 | –2.45 | 2.133 | (–8.45, 3.56) | 1 | 1 | ns |
|  | 4 vs 5 | 26.17 | 2.446 | (19.29, 33.05) | <.001 | <.001 | ** |
| PCL-C - Re-experiencing | 1 vs 2 | –1.71 | 0.429 | (–2.92, 0.51) | 0.001 | 0.008 | ** |
|  | 1 vs 3 | 3.42 | 0.44 | (2.18, 4.66) | <.001 | <.001 | ** |
|  | 1 vs 4 | 5.26 | 0.581 | (3.62, 6.89) | <.001 | <.001 | ** |
|  | 1 vs 5 | –0.77 | 0.686 | (–2.71, 1.16) | 1 | 1 | ns |
|  | 2 vs 3 | 5.13 | 0.413 | (3.97, 6.30) | <.001 | <.001 | ** |
|  | 2 vs 4 | 6.97 | 0.561 | (5.39, 8.55) | <.001 | <.001 | ** |
|  | 2 vs 5 | 0.94 | 0.669 | (–0.95, 2.82) | 1 | 1 | ns |
|  | 3 vs 4 | 1.83 | 0.569 | (0.23, 3.44) | 0.013 | 0.039 | * |
|  | 3 vs 5 | –4.20 | 0.677 | (–6.10, 2.29) | <.001 | <.001 | ** |
|  | 4 vs 5 | –6.03 | 0.776 | (–8.21, 3.85) | <.001 | <.001 | ** |
| PCL-C - Avoidance | 1 vs 2 | –3.19 | 0.503 | (–4.61, 1.78) | <.001 | <.001 | ** |
|  | 1 vs 3 | 5.31 | 0.516 | (3.86, 6.76) | <.001 | <.001 | ** |
|  | 1 vs 4 | 7.48 | 0.681 | (5.56, 9.40) | <.001 | <.001 | ** |
|  | 1 vs 5 | –3.36 | 0.805 | (–5.63, 1.10) | 0.002 | 0.012 | * |
|  | 2 vs 3 | 8.5 | 0.484 | (7.14, 9.86) | <.001 | <.001 | ** |
|  | 2 vs 4 | 10.67 | 0.658 | (8.82, 12.52) | <.001 | <.001 | ** |
|  | 2 vs 5 | –0.17 | 0.785 | (–2.38, 2.04) | 1 | 1 | ns |
|  | 3 vs 4 | 2.17 | 0.668 | (0.29, 4.05) | 0.012 | 0.036 | * |
|  | 3 vs 5 | –8.67 | 0.794 | (–10.91, 6.44) | <.001 | <.001 | ** |
|  | 4 vs 5 | –10.84 | 0.91 | (–13.40, 8.28) | <.001 | <.001 | ** |
| PCL-C - Hypervigilance | 1 vs 2 | –3.80 | 0.416 | (–4.97, 2.63) | <.001 | <.001 | ** |
|  | 1 vs 3 | 1.17 | 0.426 | (–0.03, 2.37) | 0.061 | 0.183 | ns |
|  | 1 vs 4 | 4.14 | 0.563 | (2.56, 5.73) | <.001 | <.001 | ** |
|  | 1 vs 5 | –5.82 | 0.665 | (–7.70, 3.95) | <.001 | <.001 | ** |
|  | 2 vs 3 | 4.97 | 0.4 | (3.84, 6.10) | <.001 | <.001 | ** |
|  | 2 vs 4 | 7.94 | 0.544 | (6.41, 9.47) | <.001 | <.001 | ** |
|  | 2 vs 5 | –2.03 | 0.649 | (–3.85, 0.20) | 0.018 | 0.054 | ns |
|  | 3 vs 4 | 2.97 | 0.552 | (1.42, 4.53) | <.001 | 0.001 | ** |
|  | 3 vs 5 | –7.00 | 0.656 | (–8.84, 5.15) | <.001 | <.001 | ** |
|  | 4 vs 5 | –9.97 | 0.752 | (–12.09, 7.85) | <.001 | <.001 | ** |
| Negative | 1 vs 2 | –0.83 | 0.799 | (–3.08, 1.42) | 1 | 1 | ns |
|  | 1 vs 3 | –1.74 | 0.82 | (–4.05, 0.56) | 0.338 | 1 | ns |
|  | 1 vs 4 | 3.98 | 1.083 | (0.93, 7.03) | 0.003 | 0.018 | * |
|  | 1 vs 5 | –3.32 | 1.28 | (–6.92, 0.28) | 0.096 | 0.576 | ns |
|  | 2 vs 3 | –0.91 | 0.77 | (–3.08, 1.26) | 1 | 1 | ns |
|  | 2 vs 4 | 4.81 | 1.045 | (1.87, 7.76) | <.001 | 0.006 | ** |
|  | 2 vs 5 | –2.49 | 1.248 | (–6.00, 1.02) | 0.464 | 1 | ns |
|  | 3 vs 4 | 5.72 | 1.061 | (2.74, 8.71) | <.001 | 0.001 | ** |
|  | 3 vs 5 | –1.58 | 1.262 | (–5.13, 1.97) | 1 | 1 | ns |
|  | 4 vs 5 | –7.30 | 1.446 | (–11.37, 3.23) | <.001 | <.001 | ** |
| Negative Impact on Learning - Academic Self | 1 vs 2 | –0.20 | 0.452 | (–1.09, 0.69) | 0.66 | 1 | ns |
|  | 1 vs 3 | –0.66 | 0.464 | (–1.57, 0.25) | 0.154 | 1 | ns |
|  | 1 vs 4 | 1.82 | 0.612 | (0.62, 3.02) | 0.003 | 0.018 | * |
|  | 1 vs 5 | –0.50 | 0.724 | (–1.92, 0.92) | 0.489 | 1 | ns |
|  | 2 vs 3 | –0.46 | 0.435 | (–1.32, 0.39) | 0.289 | 1 | ns |
|  | 2 vs 4 | 2.02 | 0.591 | (0.86, 3.18) | 0.001 | 0.006 | ** |
|  | 2 vs 5 | –0.30 | 0.706 | (–1.69, 1.08) | 0.669 | 1 | ns |
|  | 3 vs 4 | 2.48 | 0.6 | (1.30, 3.66) | <.001 | 0.001 | ** |
|  | 3 vs 5 | 0.16 | 0.713 | (–1.24, 1.56) | 0.822 | 1 | ns |
|  | 4 vs 5 | –2.32 | 0.818 | (–3.93, 0.72) | 0.005 | 0.03 | * |
| Negative Impact on Learning - Academic Environment | 1 vs 2 | –0.47 | 0.387 | (–1.23, 0.29) | 0.227 | 1 | ns |
|  | 1 vs 3 | –0.73 | 0.397 | (–1.51, 0.05) | 0.065 | 0.39 | ns |
|  | 1 vs 4 | 1.95 | 0.525 | (0.92, 2.98) | <.001 | 0.001 | ** |
|  | 1 vs 5 | –2.35 | 0.62 | (–3.56, 1.13) | 0.002 | 0.012 | * |
|  | 2 vs 3 | –0.27 | 0.373 | (–1.00, 0.47) | 0.475 | 1 | ns |
|  | 2 vs 4 | 2.42 | 0.506 | (1.42, 3.41) | <.001 | 0.001 | ** |
|  | 2 vs 5 | –1.88 | 0.604 | (–3.07, 0.69) | 0.002 | 0.012 | * |
|  | 3 vs 4 | 2.68 | 0.514 | (1.67, 3.69) | <.001 | 0.001 | ** |
|  | 3 vs 5 | –1.61 | 0.611 | (–2.81, 0.41) | 0.008 | 0.048 | * |
|  | 4 vs 5 | –4.30 | 0.7 | (–5.67, 2.92) | <.001 | <.001 | ** |
| Note: Post‑hoc pairwise comparisons were performed using Bonferroni correction to control the family‑wise error rate. Mean Δ = ‾χ_i_ - ‾χ_j_, represents the difference between the mean of group i and group j. SE = standard error of the mean difference. Raw ***P*** denotes the unadjusted ***P***‑value from the pairwise test; Bonf‑***P*** is the ***P***‑value after Bonferroni correction for the total number of comparisons within each measure. ** indicates statistical significance after correction (Bonf‑***P*** ≤ 0.05); ns = not significant (Bonf‑***P*** > 0.05). | | | | | | | |
